# Simultaneous reduction of radiation dose and scatter-to-primary ratio using a truncated detector and advanced algorithms for dedicated cone-beam breast CT

**DOI:** 10.1088/2057-1976/adb8f1

**Published:** 2025-03-03

**Authors:** Hsin Wu Tseng, Zhiyang Fu, Srinivasan Vedantham

**Affiliations:** 1 Department of Medical Imaging, The University of Arizona, Tucson, AZ, United States of America; 2 Department of Biomedical Engineering, The University of Arizona, Tucson, AZ, United States of America

**Keywords:** self-supervised, deep learning, breast CT, image reconstruction, dose reduction, scatter reduction

## Abstract

*Objective*. To determine the minimum detector width along the fan-angle direction in offset-detector cone-beam breast CT for multiple advanced reconstruction algorithms and to investigate the effect on radiation dose, scatter, and image quality. *Approach.* Complete sinograms (*m* × *n* = 1024 × 768 pixels) of 30 clinical breast CT datasets previously acquired on a clinical-prototype cone-beam breast CT system were reconstructed using Feldkamp-Davis-Kress (FDK) algorithm and served as the reference. Complete sinograms were retrospectively truncated to varying widths to understand the limits of four image reconstruction algorithms—FDK with redundancy weighting (FDK-W), compressed-sensing based FRIST, fully-supervised MS-RDN, and self-supervised AFN. Upon determining the truncation limits, numerical phantoms generated by segmenting the reference reconstructions into skin, adipose, and fibroglandular tissues were used to determine the radiation dose and scatter-to-primary ratio (SPR) using Monte Carlo simulations. *Main results.* FDK-W, FRIST, and MS-RDN showed artifacts when *m* < 596, whereas AFN reconstructed images without artifacts for *m* > = 536. Reducing the detector width reduced signal-difference to noise ratio (SDNR) for FDK-W, whereas FRIST, MS-RDN and AFN maintained or improved SDNR. Reference reconstruction and AFN with *m* = 536 had similar quantitative measures of image quality. *Significance.* For the 30 cases, AFN with *m* = 536 reduced the radiation dose and SPR by 37.85% and 33.46%, respectively, compared to the reference. Qualitative and quantitative image quality indicate the feasibility of AFN for offset-detector cone-beam breast CT. Radiation dose and SPR were simultaneously reduced with a 536 × 768 detector and when used in conjunction with AFN algorithm had similar image quality as the reference reconstruction.

## 1. Introduction

X-ray cone-beam computed tomography (CBCT) offers isotropic internal anatomical structures over a large area in any orientation in a rapid single scan. Although the performance of CBCT with a large flat-panel detector (FPD) has advanced progress in the past decades, CBCT is still considered a tool only for high-contrast features. Recent developments in technologies of detector and image reconstruction algorithms make diagnostic imaging tasks of low-contrast resolution possible. Dedicated cone-beam breast computed tomography (CBBCT) is an emerging modality for breast cancer imaging (O’Connell *et al* 2014, Vedantham and Karellas 2018). Unlike digital mammography (DM) and digital breast tomosynthesis (DBT), CBBCT does not need the physical compression of the breast. Besides, the masking effect that could cause suspicious areas due to tissue overlap is mitigated by CBBCT. In addition, the sensitivity of CBBCT in the diagnostic setting has been shown to be higher than DM in a reader study (Cole *et al* 2015). CBBCT also improves over DBT by overcoming various artifacts associated with limited-angle tomography (Sujlana *et al* 2019). Currently, CBBCT scanners have 300 or 500 projections in a circular trajectory (Lindfors *et al* 2008, O’Connell *et al* 2010). Recently, a CBBCT system incorporating a high-resolution laterally-shifted detector has been evaluated (Trinate *et al* 2023, Tseng *et al* 2023a). However, like other CBCT systems, CBBCT systems using large FPDs suffer from scattered radiation. Scatter events may cause cupping artifacts and reduced image contrast and lead to inaccurate CT numbers (Hounsfield Units) in reconstructed images. Another drawback is that the visualization of microcalcifications degrades when the radiation dose is reduced to a level similar to that of 2-view mammography (Lindfors *et al* 2008). The dose metric for DM, DBT, and CBBCT is the mean glandular dose (MGD). The MGD is related to the energy deposited on fibroglandular tissues which are at risk for developing breast cancer (Hammerstein *et al* 1979). The MGD is limited to 3 mGy by the Mammography Quality Standards Act (MQSA) for the cranio-caudal (CC) view of a phantom that models a breast of average size (FDA US 2002). The MGD of standard 2-view screening mammography reported by the ACRIN-DMIST trial is about 4.15–4.98 mGy (Hendrick *et al* 2010). Hence, the MGD ranges from 3 to 6 mGy for using CBBCT as a breast cancer screening tool because CBBCT uses a single scan instead of two standard views used with DM and DBT. For CBBCT systems used for non-contrast diagnostic imaging, the MGD is in the range of 7.2–12.5 mGy (O’Connell *et al* 2010, Vedantham *et al* 2013, Wienbeck *et al* 2017). Hence, reducing the scatter to improve microcalcification visibility (Ghazi 2020) and lowering the radiation dose is essential to translate CBBCT to be a screening modality.

Several scatter reduction and correction methods have been developed for breast CT imaging. Well-known approaches, such as using tungsten plates (Sechopoulos 2012, Ramamurthy *et al* 2016) and narrow beams (Ghazi *et al* 2023), provide significant suppression of cupping artifacts. A library-based correction method that only requires breast diameter as an input precomputes a scatter library using simplified breast models of various sizes via Monte Carlo (MC) simulations and efficiently reduces scatter (Shi *et al* 2016). Forward-projection model uses prior images generated by segmented CBBCT images followed by a Fourier transform-based algorithm to remove scatter (Shi *et al* 2017). A U-Net-based deep learning (DL) model trained by MC-generated projections developed to estimate the scatter signal in CBBCT can correct cupping artifacts (Pautasso *et al* 2023). On the other hand, there were many advanced technologies aimed at reducing the radiation dose in CBBCT. Advanced reconstruction algorithms such as compressed-sensing (Sidky and Pan 2008, Bian *et al* 2014, Tseng *et al* 2020b) and deep-learning methods (Fu *et al* 2020, Xie *et al* 2020) are common approaches to reducing radiation doses. The feasibility of using protocols such as short scans, sparse views, and shorter pulse widths (Tseng *et al* 2020a, 2023a, 2023b, ) was also demonstrated as another strategy to reduce the dose for CBBCT. However, none of the above approaches can reduce the SPR and radiation dose simultaneously.

**Figure 1.**
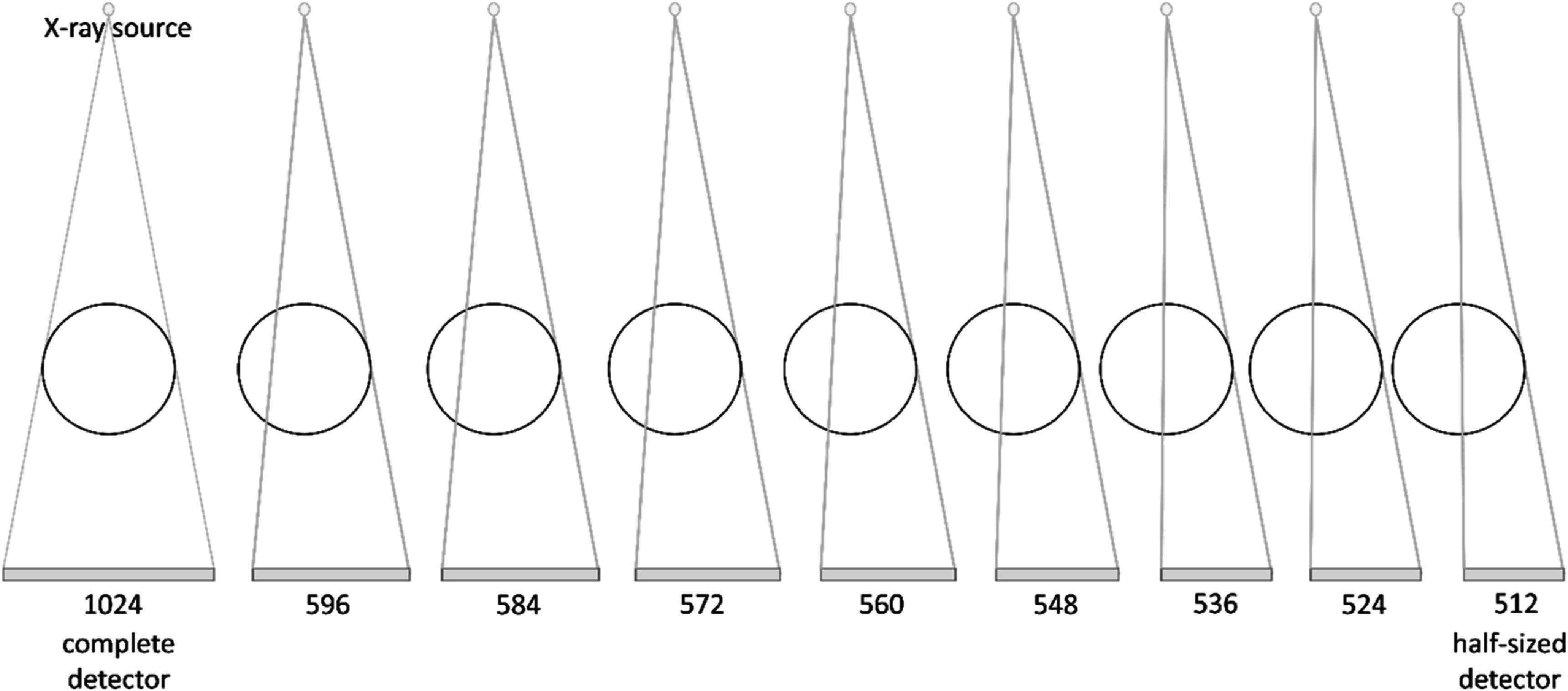
The number of pixels in the detector along the fan-angle direction showing the truncation scheme. The left-most panel is the original/reference dimension of the detector used in the system.

In this study, we proposed to use a nearly half-sized FPD with a narrower beam to simultaneously reduce the scatter and radiation dose. The scatter effect has been demonstrated to be substantially reduced when the dimension of the FPD along the fan-angle direction is truncated to half the size (Mettivier *et al* 2012). Ideally, the dose will be reduced up to 50% for a homogeneous breast when half the x-ray beam is blocked. A more accurate dose estimate can be obtained using MC simulations. The most challenging part of this approach is to reconstruct the breast from the projection missing approximately half of the information at every projection (view) angle. For fan-beam geometry, the image can be successfully reconstructed (Rutt and Fenster 1980). However, this approach is not trivial for cone-beam geometry. The traditional image reconstruction algorithm, Feldkamp-Davis-Kress (FDK) (Feldkamp *et al* 1984), with weighting schemes (Cho *et al* 1996, Wang 2002, Schaefer *et al* 2011) has been developed and applied in our CBBCT system with a laterally-shifted truncated detector (Vedantham *et al* 2020, Trinate *et al* 2023). FDK with weighting schemes leverages data redundancy, i.e., information from complementary projections to estimate missing information. However, this method might fail when the number of detector pixels, *m*, along the fan-angle direction is too small (figure 1). To address this issue, we investigated advanced algorithms including the recently developed a self-supervised deep learning (SSDL) algorithm, attenuation field network (AFN) (Fu *et al* 2024). This study evaluates the performance and limits of these algorithms in terms of the number of pixels needed along the fan-angle direction in the detector for artifact-free reconstruction and on image quality. Once the number of pixels needed were determined for each algorithm, MC simulations were conducted to determine the MGD and scatter-to-primary ratio (SPR) from which the percent reduction compared to the complete detector were determined. Thus, this article has two distinct synergistic parts: (1) investigating image reconstruction algorithms to identify the minimum detector size using retrospective clinical datasets, and (2) conducting Monte Carlo simulations to determine the radiation dose and SPR redcutions achieved with minimum detector size identified from the first part.

## 2. Methods and materials

### 2.1. Clinical data

In this retrospective study, sinograms were obtained from 30 women who were part of an institutional review board-approved, HIPPA-compliant clinical study (clinicaltrials.gov: NCT01090687). Since the image noise in low-dose acquisitions degrade the visualization of microcalcifications in CBBCT, most of the breast volumes (29/30) were selected to contain calcifications. For the cohort included in the study, the mean ± standard deviation of the chest-wall diameter, chest-wall to nipple length, and fibroglandular weight fraction were $12.26\pm 2.68$, $10.4\pm 2.75$, and $0.20\,\pm 0.16$, respectively.

All projection datasets were acquired using a pre-FDA approval prototype dedicated CBBCT system (Koning Corp, Norcross, GA, USA). The CBBCT scanner used a flat-panel detector (PaxScan 4030CB, Varex Imaging, Salt Lake City, UT, USA) with dimensions of 40 cm and 30 cm along the fan-angle and cone-angle directions, respectively, which has a field of view (FOV) sufficient to cover the breast in its entirety. The detector was operated in a 2 × 2 binned mode, resulting in 0.388 mm × 0.388 mm detector pixels. The x-ray tube (RAD-71SP, Varex Imaging, Salt Lake City, UT, USA) was operated in pulse mode (8 ms), and the x-ray spectrum was 49 kVp (1^st^ half-value layer thickness: 1.4 mm of Al). There were 300 projections acquired over 360 degrees for each sample, and the scan time was 10 s. The source-to-detector distance was 898 mm, and the distance between the source and the axis of rotation (AOR) was 650 mm.

### 2.2. Emulation of the offset incomplete detector CBBCT system

The complete sinogram has 1024 × 768 pixels along the fan- and cone-angle directions. To emulate the sinograms acquired from an incomplete detector with a narrow beam, we retrospectively truncated the projection along the fan-angle direction at each view angle into 596, 584, 572, 560, 548, 536, 524, and 512 pixels (figure 1), while maintaining the same cone-angle extent of 768 pixels, to investigate the performance limits of the image reconstruction algorithms.

### 2.3. Image reconstruction algorithms

All algorithms investigated in this study used projection data from human subjects. All algorithm used full-scan data encompassing 360° acquisition. The complete sinograms (1024 × 768 pixels) were reconstructed using the FDK algorithm. The truncated projections (*m* × 768 pixels), where *m* ranges 596-512 pixels were reconstructed using each of the following algorithms.

#### 2.3.1. FDK with weighting schemes

The truncated detector can be reconstructed by FDK with appropriate weighting schemes (Cho *et al* 1996, Wang 2002, Schaefer *et al* 2011) due to redundancy. We have demonstrated this method before (Vedantham *et al* 2020) and have shown that there is no significant difference in the statistical results among the three common weighting functions that were proposed by Cho (Cho *et al* 1996), Schaefer (Schaefer *et al* 2011), and Wang (Wang 2002). These schemes leverage the information from complementary projections to estimate the missing information. The concept of weighting function proposed by above authors is to smooth the truncated edge in the sinogram. Hence, cosine, sine and sinc functions have been proposed to provide a smoother transition along the edge. For further details on the implementation of these weighting schemes and their evaluation, we refer to our prior publication (Vedantham *et al* 2020). For the rest of the paper, we used FDK-W for FDK using Wang’s weighting function. In this study, the reconstruction voxel size was 0.2734 mm and the reconstructed volume had 1024 × 1024 × Z voxels (Z depends on the length from the chest wall to the nipple).

#### 2.3.2. FRIST algorithm

The previously developed algorithm, Fast, total variation-Regularized, Iterative, Statistical reconstruction Technique (FRIST) (Tseng *et al* 2020b), has been demonstrated to have better image quality than FDK in an offset or truncated detector (Tseng *et al* 2022). We have previously shown that using FDK as an initial value rather than zero is more efficient with complete detectors (Tseng *et al* 2020b). Thus, in the study of incomplete detectors here, using FDK-W as an initial value was considered more practical in terms of computational time. Ideally the number of main iterations and total-variation (TV) steps should be dependent on the voxel size of the object, detector pixels, geometry of the imaging system, and scanning protocols. To simplify the process of determing the parameters, approximations were used based on our prior studies. The convergence analysis and the number of TV iterations were calibrated before based on both experienced medical physicists and mathematical approaches for *m* = 768 and 640. Since the dimension, *m*, in this study was less than 640, more TV iterations were expected for denoising. Hence, we used the number of TV iterations from the area under the image power spectrum (AUP) method (table 1 in Tseng *et al* 2022) for *m* = 640 in this study. The number of TV iterations is summarized in table 1.

**Table 1.** Summary of the number of TV iterations for FRIST in this study.

Breast size (chest-wall diameter)	Number of TV iterations
Large	10
Medium	10
Small	15

#### 2.3.3. Supervised training deep-learning approach

Our previously developed supervised learning approach, MS-RDN (Fu *et al* 2020), requires external training datasets for the training processes. The architecture of MS-RDN has multi-slice inputs processed by a shared 2D convolutional layer. The generated 3D spatial features propagate through both high- and low-resolution branches. These learned high- and low-resolution features are then summed using a trainable weighting factor. The output convolutional layer reconstructs multi-slice outputs from the fused feature maps. We provided 20 samples for training and 1 sample for validation. Considering that on average there are 450 slices per sample, approximately 9000 slices were used for training. All 21 samples had microcalcifications. Due to computational expense, *m* = 596 alone was used for the training. The input images were reconstructed from truncated detectors and the target datasets were images reconstructed from complete detectors.

#### 2.3.4. Self-supervised deep-learning approach

Our recently developed deep-learning (DL) algorithm (Fu *et al* 2024) is self-supervised, which does not need external data for the training procedures. The method is called attenuation field network (AFN). This algorithm has demonstrated that it can reconstruct sinograms from an incomplete detector (Fu *et al* 2023, 2024). Inspired by the ability of sinogram inpainting by AFN, we applied this technique in this study. This study differs from the prior work in that the acquisition is a full scan with the dimension of the detector fan-angle direction is nearly 512 pixels.

A minimum training sample in the training procedure of the AFN technique is a ray propagating from the x-ray source ***s*** to a detector pixel ***d***. Multiple samples are obtained through the ray $\mathop{{\boldsymbol{sd}}}\limits^{\longrightarrow}$, and they can be represented as ${{\boldsymbol{t}}}_{i}={\boldsymbol{s}}+{\alpha }_{i}\left({\boldsymbol{d}}-{\boldsymbol{s}}\right)$, $0< {\alpha }_{i}\,< {\alpha }_{i+1}< 1,\forall i$. Therefore, the attenuation coefficients of these samples are integrated to provide the estimated projection which can be expressed as

\begin{eqnarray*}\hat{{\boldsymbol{p}}}\left({\boldsymbol{d}}\right)=\displaystyle \displaystyle \sum _{i}{h}_{{\mathrm{\Theta }}}\left({{\boldsymbol{t}}}_{i}\right)\left|{{\boldsymbol{t}}}_{i+1}-{{\boldsymbol{t}}}_{i}\right|,\end{eqnarray*}

where $\hat{{\boldsymbol{p}}}\left({\boldsymbol{d}}\right)$ is the estimated projection and ${{\mathrm{h}}}_{{\mathrm{\Theta }}}\left(\cdot \right)$ denotes an AFN parameterized by ${\mathrm{\Theta }}$. The error between the estimated projection $\hat{{\boldsymbol{p}}}\left({\boldsymbol{d}}\right)$ and the real projection ${\boldsymbol{p}}\left({\boldsymbol{d}}\right)$ serves as the training loss to optimize the network’s representation:

\begin{eqnarray*}\mathop{{\mathrm{argmin}}}\limits_{{\mathrm{\Theta }}}\displaystyle \displaystyle \sum _{{\boldsymbol{d}}}{\parallel \hat{{\boldsymbol{p}}}\left({\boldsymbol{d}}\right)-{\boldsymbol{p}}\left({\boldsymbol{d}}\right)\parallel }^{2},{\boldsymbol{d}}\in {\mathbb{D}},\end{eqnarray*}

where ${\mathbb{D}}$ denotes the set of coordinates of all of the acquired projection data. A trained AFN is a three-dimensional (3D) representation of the attenuation coefficients of the object. When the same imaging geometry (as that of the acquired data) is emulated, AFN can inpaint data that are unacquired. The inpainted projection data are spliced with the acquired projection data to yield complete hybrid projection data, which can be subsequently reconstructed by any desired reconstruction methods. In this study, the complete hybrid data were reconstructed by FDK.

### 2.4. Scatter-to-primary ratio (SPR) and mean glandular dose (MGD) estimate

**Figure 2.**
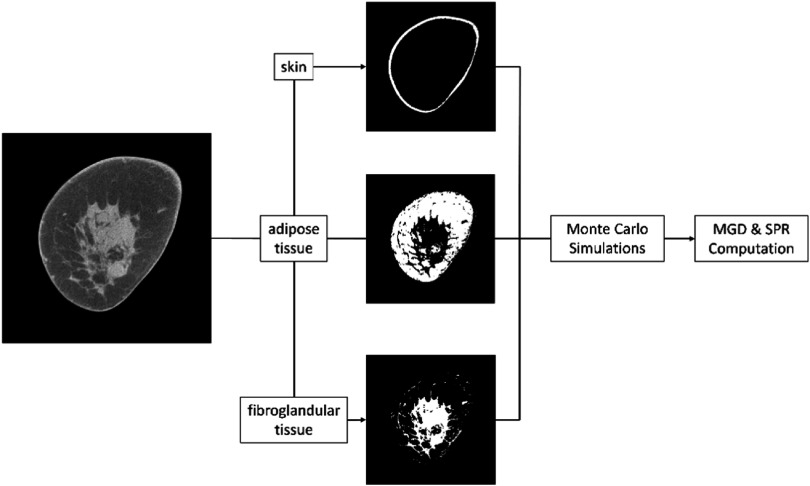
Segmentation of the image. The FDK image of each clinical sample was segmented into air (black area), skin, adipose, and fibroglandular tissue followed by the Monte Carlo simulations and computations of mean glandular dose (MGD) and scatter-to-primary ratio (SPR).

For Monte Carlo simulations to estimate mean glandular dose (MGD) and scatter-to-primary ratio (SPR), we need to generate patient-specific numerical models of each breast. For this, we used FDK reconstruction using the complete sinogram to obtain the 3D breast volume. Each 3D breast volume was segmented into the air, skin, adipose, and fibroglandular tissues (figure 2) using methods described in prior works (Vedantham *et al* 2012b, Shi *et al* 2013). Each segmented tissue type was assigned its physical properties including mass, density, and energy-depenent attenuation coefficients to provide 3D numerical models.

**Figure 3.**
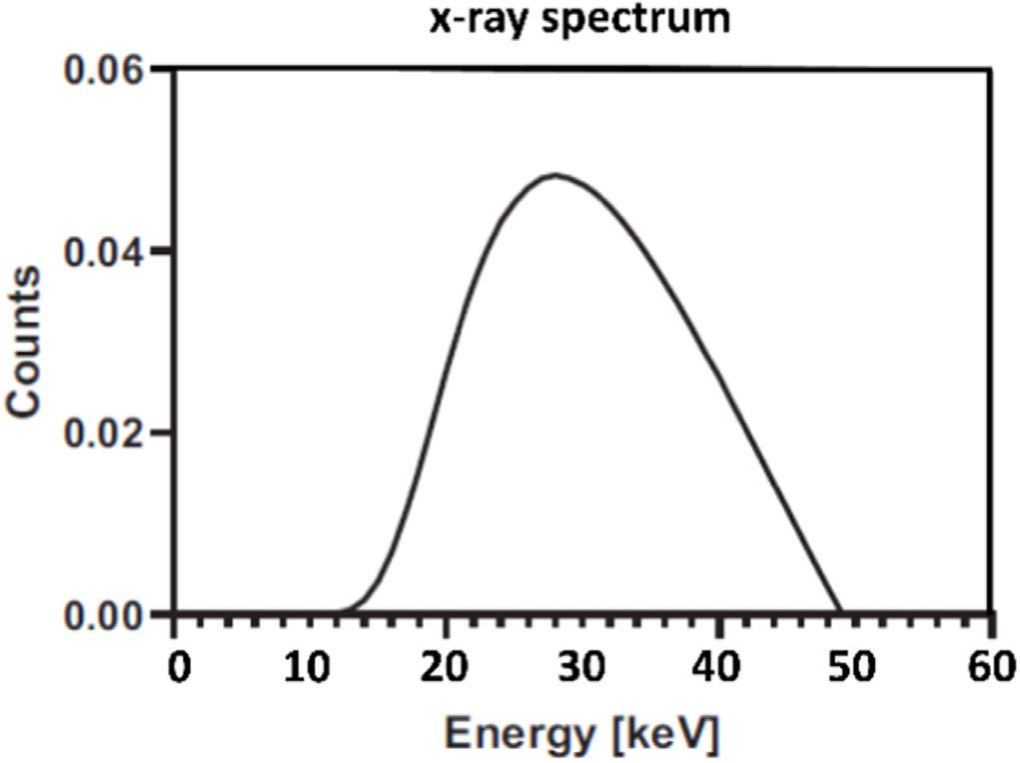
Tungsten-anode 49 kV x-ray spectrum normalized to unit area.

The 3D numerical models were used in Monte Carlo (MC) simulations, which were performed using GATE 8.0 (GEANT4-based). The MC simulations were performed for estimating the SPR and radiation dose not for imaging. $3\times {10}^{10}$ x-ray photons were sampled from the x-ray spectrum (49 kV) that matched the half-value layer (1.39 mm of Al) of the CBBCT system used for acquisition (figure 3), and distributed over 30 equiangular projections (12° apart over 360°). The x-ray focal spot was simulated as a 0.3 mm × 0.3 mm square. The implementation of the MC simulations was verified in our previous work (Tseng *et al* 2021), showing that 30 projections and ${10}^{6}$ photons can achieve a coefficient (COV) of less than 0.7% in the amount of energy deposited. Prior studies have also shown tracking $1\times {10}^{6}$ x-ray photons is sufficient for dose estimation (Vedantham *et al* 2012a). For scatter estimation, $1.2\times {10}^{7}$ photons per energy bin (Shi *et al* 2016), and $2\times {10}^{8}$ photons (Pautasso *et al* 2023) distributed over 12 equiangular projections ($30^\circ $ apart over $360^\circ $) were found to be sufficient in prior studies. We used $3\times {10}^{10}$ photons, which is two orders of magnitude higher, and finer angular sampling ($12^\circ $ apart over $360^\circ $) to provide a more robust estimate.

To calculate the mean glandular dose (MGD; unit = mGy) of a real breast, we calculate the normalized glandular dose coefficient, $Dg{N}^{CT}$ (mGy/mGy), of the heterogeneous model followed by multiplying the air kerma (mGy). The heterogeneous $Dg{N}^{CT}$ is expressed as (Sechopoulos *et al* 2012)

\begin{eqnarray*}Dg{N}_{hete}^{CT}=\displaystyle \frac{{E}_{g,dep}}{{n}_{g}{m}_{g}\displaystyle {\sum }_{E}{\mathrm{\Phi }}\left(E\right){\mathrm{\Theta }}\left(E\right)}\end{eqnarray*}

where ${E}_{g,dep}$ is the total energy deposited in all fibroglandular voxels, ${n}_{g}$ is the total number of fibroglandular tissue voxels, ${m}_{g}$ is the mass of fibroglandular tissue voxels, ${\mathrm{\Phi }}\left(E\right)$ is the x-ray fluence function of energy, *E*, and ${\mathrm{\Theta }}\left(E\right)$ is the fluence-to-air kerma conversion factor at energy *E*.

### 2.5. Quantitative image quality evaluations

Traditional image quality metrics in terms of first (mean value) and second (variance) statistics, signal-difference to noise ratio (SDNR), and the full-width at half-maximum (FWHM) of calcifications were computed. A 32 pixel × 32 pixel region of interest (ROI) was extracted from both fibroglandular and adipose tissues to estimate the mean $\left(\bar{\mu }\right)$ and variance $\left({\sigma }^{2}\right)$ of the signal and background, respectively. The mean and variance of fibroglandular and adipose ROIs were considered here for verifying the pixel value that is related to the CT number was not deviated when the variance reduced by the advanced algorithms. The same ROIs were also used to estimate the SDNR which can be expressed as:

\begin{eqnarray*}SDNR=\displaystyle \frac{\left|\overline{{\mu }_{G}}-\overline{{\mu }_{A}}\right|}{\sqrt{\displaystyle \frac{1}{2}\left({\sigma }_{G}^{2}+{\sigma }_{A}^{2}\right)}}\end{eqnarray*}

The subscription, *G* and *A*, indicate fibroglandular and adipose tissue region, respectively. Thus, the computed SDNR is between adipose and fibroglandular tissues and not between background and lesion. The rationale for determining the FWHM of calcifications is to provide a measure of spatial resolution. The advanced image reconstruction algorithms studied, while reducing image noise, could also result in image blur and hence degrade the FWHM of calcificaitons. Hence, the FWHM of calcifications were determined along two orthogonal directions: the mediolateral (ML) and the superior-inferior (SI) directions to provide an estimate the spatial resolution. As noted earlier, 29 of the 30 breast volumes considered had calcifications.

## 3. Results

### 3.1. FDK algorithm with weighting schemes

**Figure 4.**
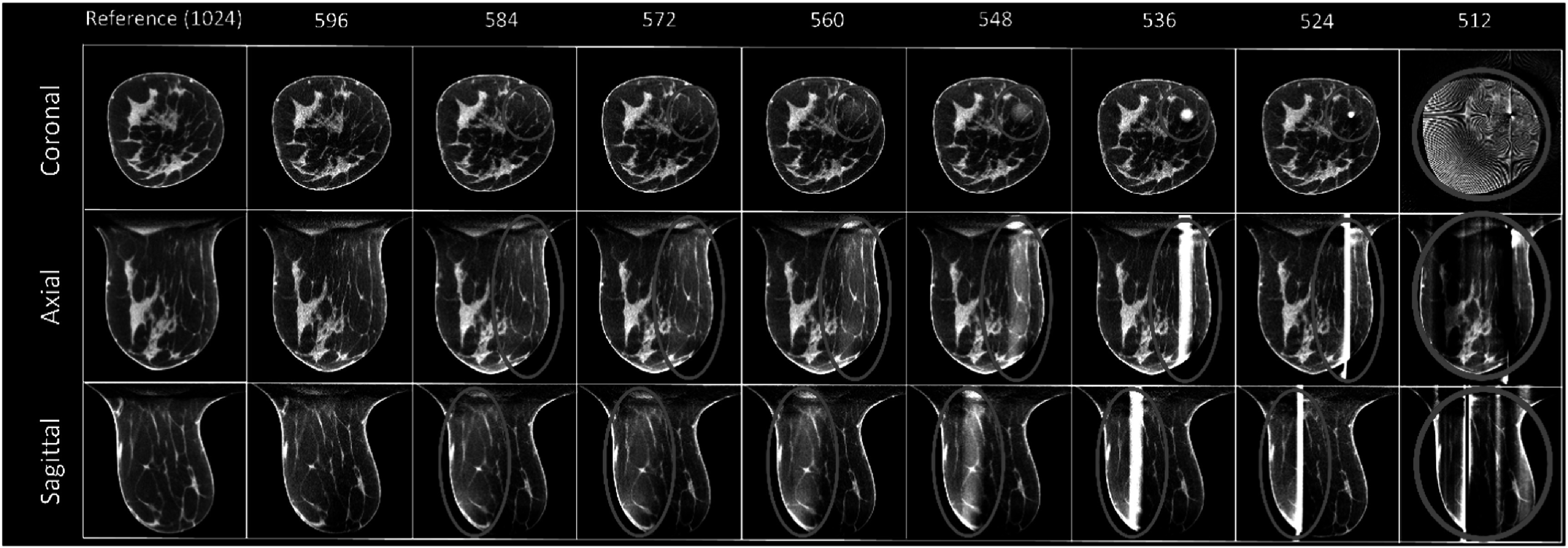
FDK reference and FDK-W images (central slice; magnified). From top to bottom: coronal, axial, and sagittal view. From left to right are the FDK-W images reconstructed with varying number of detector pixels along the fan-angle direction. The reference FDK images use a complete detector (1024 × 768 pixels). All images were displayed as a 5-slice average with a window of [0.2, 0.3] ${{\mathrm{cm}}}^{-1}$.

This method fails when the truncated area is more than 42%. For a complete detector with 1024 × 768 pixels, artifacts can be observed when the fan-angle direction dimension is truncated from 1024 to 584 pixels (figure 4). The shading artifact can be seen for 584 pixels, and this shading area became smaller and brighter when truncation increased. The center of this artifact was located at the center of the reconstructed FOV. Please note that the breast is slightly off-center due to positioning error by the technologist. In this study, *m* = 596 was considered as the performance limit of the FDK-W algorithm.

### 3.2. FRIST algorithm

**Figure 5.**
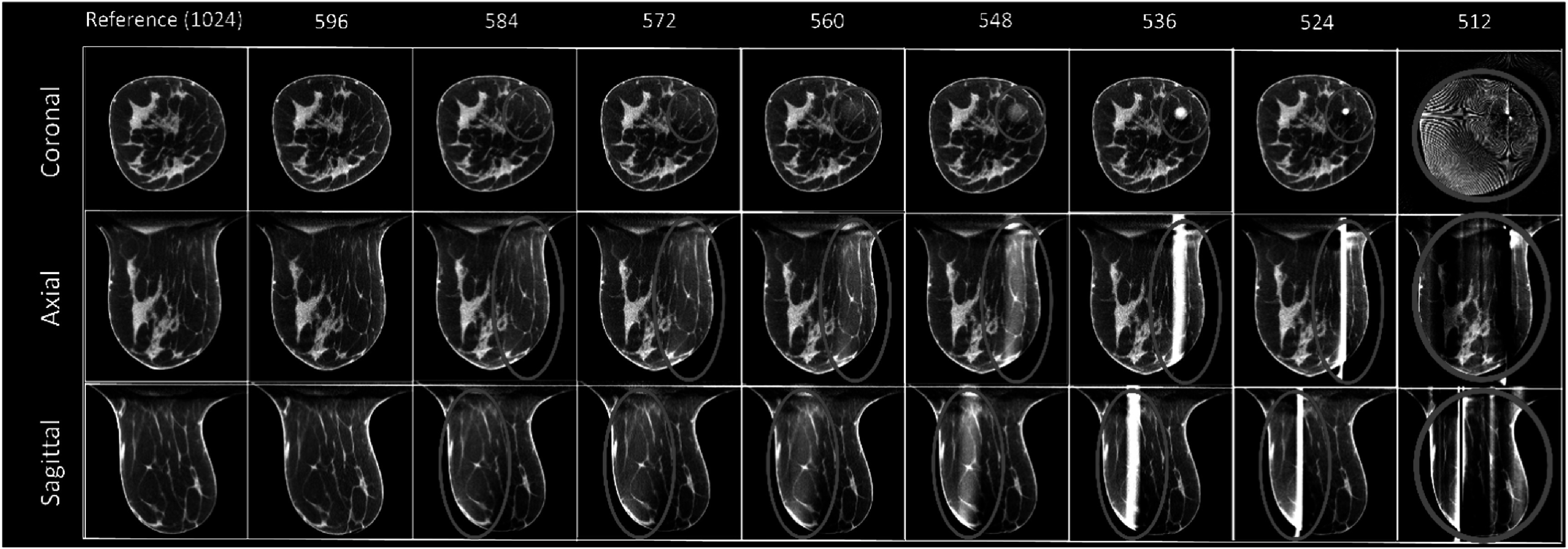
FDK reference and FRIST images (central slice; magnified). From top to bottom: coronal, axial, and sagittal view. From left to right are the FIRST images reconstructed with varying number of detector pixels along the fan-angle direction. The reference FDK images use a complete detector (1024 × 768 pixels). All images were displayed as a 5-slice average with a window of [0.2, 0.3] ${{\mathrm{cm}}}^{-1}$.

By using the total-variation regularization, the less noisy results than FDK can be seen in FRIST reconstructed images (figure 5). However, like FDK, FRIST images had brighter and circular artifacts around the center of FOV when *m* = 584 and had obvious cylindrical artifacts at the center of FOV when *m* = 536. Thus, we used *m* = 596 for FRIST images for the quantitative image quality evaluations compared with the results of other approaches.

### 3.3. Fully-supervised deep-learning MS-RDN approach

**Figure 6.**
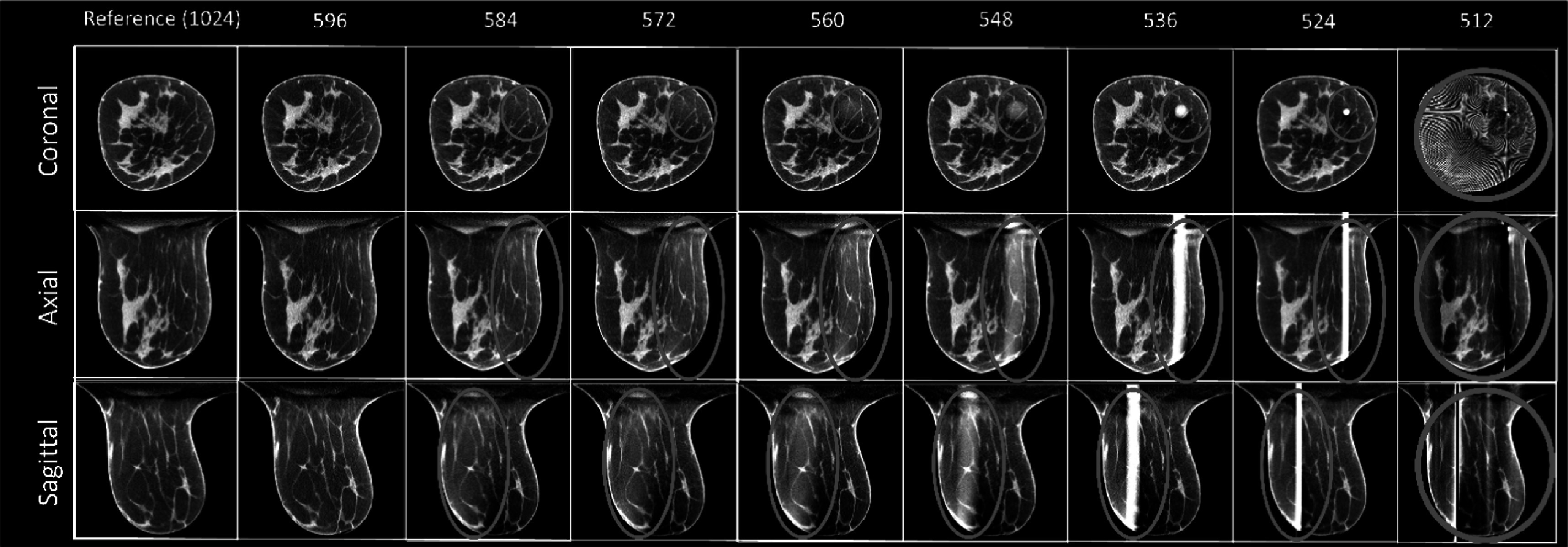
FDK reference and MS-RDN images (central slice; magnified). From top to bottom: coronal, axial, and sagittal view. From left to right are the MS-RDN images reconstructed with varying number of detector pixels along the fan-angle direction. The reference FDK images use a complete detector (1024 × 768 pixels). All images were displayed as a 5-slice average with a window of [0.2, 0.3] ${{\mathrm{cm}}}^{-1}$.

The results of the MS-RDN algorithm were similar to that of FDK and FRIST (figure 6). All of them have shown artifacts (cylindrical and brighter areas) around the center of the reconstructed FOV. Ideally the dimension of the truncated sinogram, *m*, of training samples and that of the testing image should be consistent. We realized that the MS-RDN is a post-processing DL algorithm on the image domain, which implies that MS-RDN would not be able to inpainting the missing sinogram. In addition, the training process is very time consuming. Since the artifacts can be observed when the *m* = 586, we used *m* = 596 as an approximation for MS-RDN image reconstructions for further quantitative image quality evaluations in the rest of the study. Like FRIST, MS-RDN showed less noise in images than FDK and AFN.

### 3.4. Self-supervised deep-learning AFN approach

**Figure 7.**
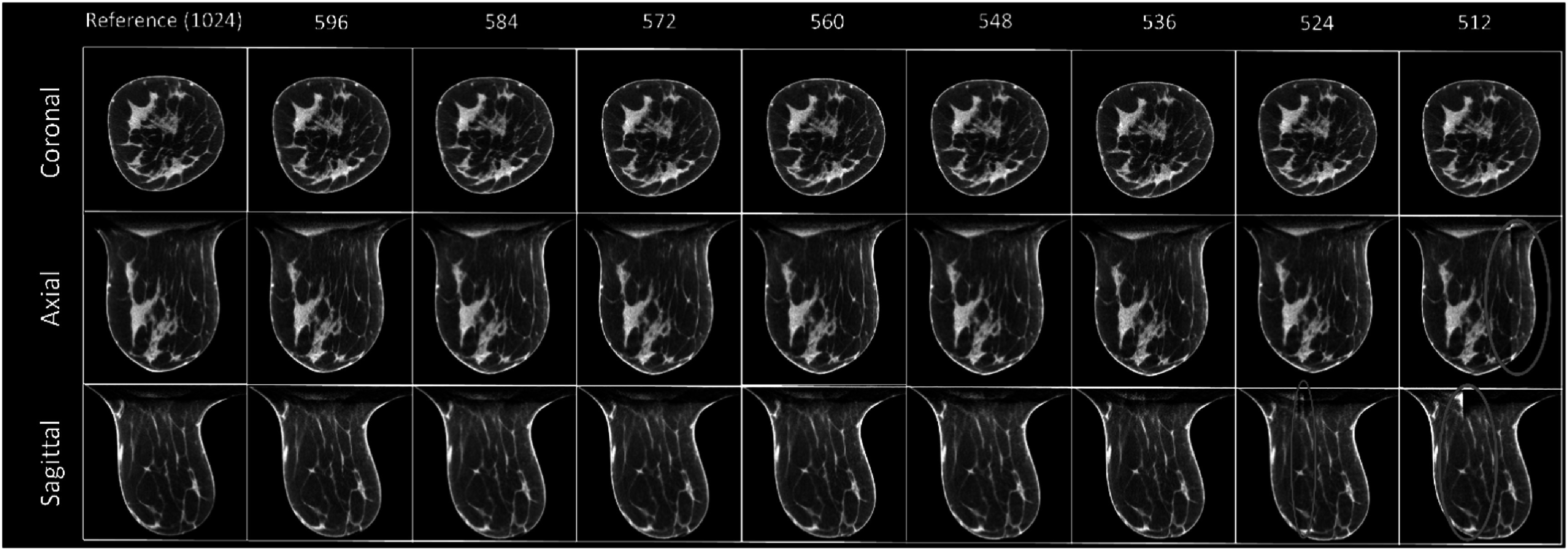
FDK reference and AFN images (central slice; magnified). From top to bottom: coronal, axial, and sagittal view. From left to right are the ANF images reconstructed with varying numbers of detector pixels along the fan-angle direction. The reference FDK images use a complete detector (1024 × 768 pixels). All images were displayed as a 5-slice average with a window of [0.2, 0.3] ${{\mathrm{cm}}}^{-1}$.

**Figure 8.**
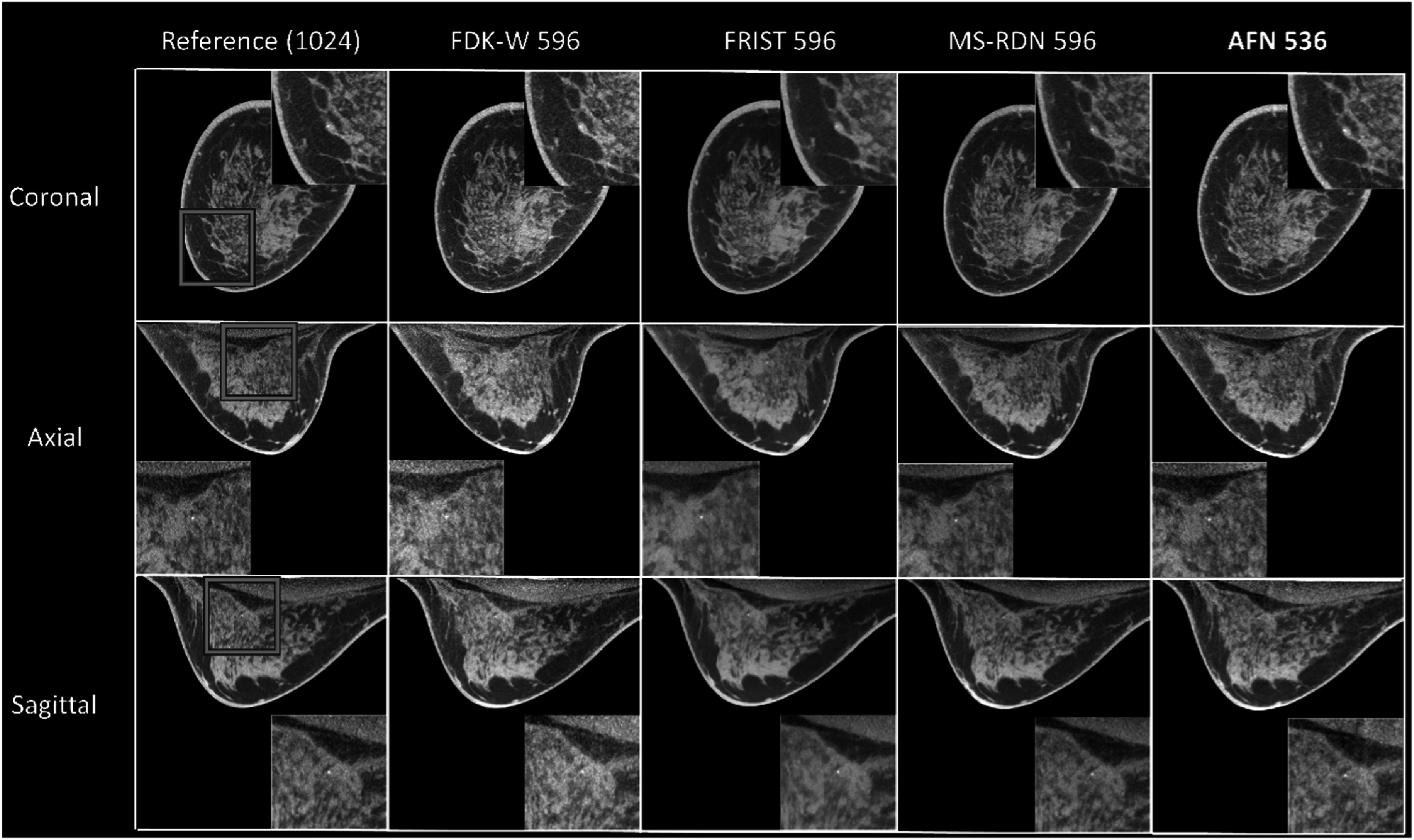
An example case with calcification. Blue squares shows the ROI surrounding the calcification. The insert in each panel shows the ROI. From top to bottom: coronal, axial, and sagittal view. The reference FDK images were obtained with a complete detector (1024 × 768 pixels). The FDK-W, FRIST, and MS-RDN images were reconstructed from a truncated detector (596 × 768 pixels). The AFN images were obtained with a truncated detector (536 × 768 pixels). All images were displayed as a 5-slice maximum intensity projection (MIP) with a window of [0.2, 0.3] ${{\mathrm{cm}}}^{-1}$.

The recently developed self-supervised deep learning method, AFN, can handle this challenge better. There was no artifact observed even when the number of pixels along the fan-angle direction was decreased to 536 (figure 7). However, some samples among the thirty clinical data were found artifacts when the number of pixels, *m*, was less than 536. Conservatively, the 536-pixel detector was considered as the performance limit of the AFN algorithm. The calcifications can be visualized clearly in AFN images reconstructed from the 536 × 768 detector (figure 8) compared with other reconstruction algorithms using 596 × 768 detector. Visually, there is no reduction in sharpness with AFN.

### 3.5. Quantitative image quality evaluations

For the quantitative image quality evaluations of truncated detectors, 596 × 768 detectors were used for FDK, FRIST, MS-RDN, and AFN algorithms to ensure that there were no artifacts. For the AFN image reconstruction algorithm, a 536 × 768 detector was studied as well.

**Figure 9.**
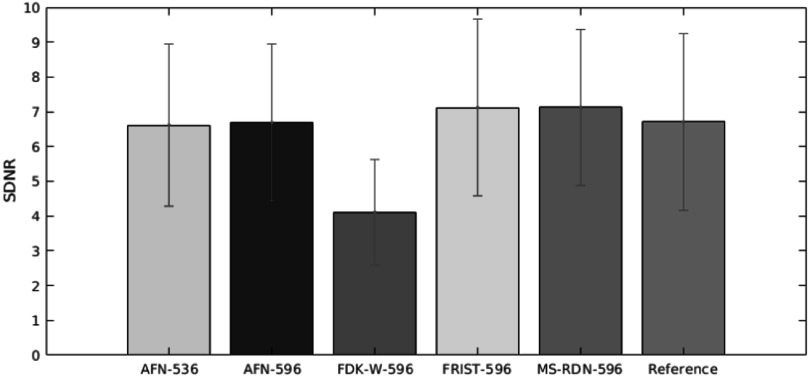
The summary of SDNR. Green: AFN images reconstructed from the 536 × 768 detector. Blue: AFN images reconstructed from the 596 × 768 detector. Red: FDK-W images reconstructed from the 596 × 768 detector. Cyan: 596-detector using FRIST. Magenta: MS-RDN algorithm with 596-detector. Dark green (The reference): FDK images reconstructed from the 1024 × 768 detector.

The quantitative image quality was measured based on the mean and variance value of adipose and fibroglandular tissues, signal difference-to-noise ratio (SDNR), and full-width at half maximum (FWHM; mm) of microcalcifications at the mediolateral (ML) direction and the superior-inferior (SI) direction.. For mean values of adipose and fibroglandular tissue, there was no significant difference among all algorithms. The FDK-W had the highest variance for adipose and fibroglandular tissues. FRIST and MS-RDN improved the variance. The variance in AFN images (596 and 536) was similar to that of reference images. MS-RDN had the lowest calcification resolution in terms of FWHM in ML and SI directions. The SDNR values are summarized in figure 9. The image quality is summarized in table 2.

**Table 2.** Summary of image quality of different image reconstruction algorithms including traditional FDK, FDK-W, FRIST, MS-RDN, and AFN algorithms.

	Mean of adipose tissue $\left({{\mathrm{cm}}}^{-1}\right)$	Variance of adipose tissue $\left(\times {10}^{-4}\,{{\mathrm{cm}}}^{-2}\right)$	Mean of fibroglandular tissue $\left({{\mathrm{cm}}}^{-1}\right)$	Variance of fibroglandular tissue $\left(\times {10}^{-4}\,{{\mathrm{cm}}}^{-2}\right)$	SDNR	FWHM ML (mm)	FWHM SI (mm)
Reference (FDK)	$0.2318\pm 0.0127$	$0.6600\pm 0.6766$	$0.2861\pm 0.0195$	$0.9550\pm 0.7171$	$6.7102\pm 2.5353$	$1.6687\pm 0.5714$	$1.6687\pm 0.7473$
596-detector (FDK-W)	$0.2321\pm 0.0143$	$1.7759\pm 1.1845$	$0.2866\pm 0.0217$	$2.2608\pm 1.1822$	$4.1232\pm 1.5087$	$1.6593\pm 0.4971$	$1.6687\pm 0.7711$
596-detector (FRIST)	$0.2326\pm 0.0145$	$0.4942\pm 0.4883$	$0.2892\pm 0.0214$	$0.8148\pm 0.7668$	$7.1196\pm 2.5346$	$1.6687\pm 0.5623$	$1.6404\pm 0.4870$
596- detector (MS-RDN)	$0.2301\pm 0.0133$	$0.6051\pm 0.5466$	$0.2868\pm 0.0188$	$0.8878\pm 0.6026$	$7.1300\pm 2.2367$	$1.7064\pm 0.6567$	$1.7158\pm 0.7104$
596-detector (AFN)	$0.2319\pm 0.0127$	$0.6435\pm 0.3710$	$0.2894\pm 0.0191$	$1.0376\pm 0.7347$	$6.6858\pm 2.2494$	$1.6497\pm 0.4338$	$1.6310\pm 0.4842$
536-detector (AFN)	$0.2331\pm 0.0129$	$0.6488\pm 0.6829$	$0.2884\pm 0.0196$	$1.0531\pm 0.8306$	$6.6170\pm 2.3151$	$1.6404\pm 0.5653$	$1.6310\pm 0.7581$

### 3.6. Scatter-to-primary ratio (SPR) and mean glandular dose (MGD) estimates

**Figure 10.**
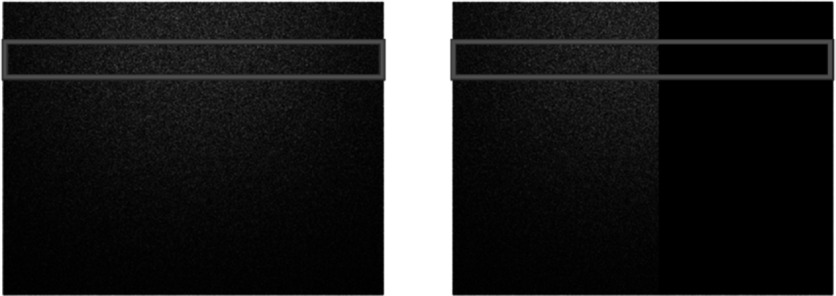
The simulated scatter-only images averaged by all projection views. Left: complete detector (m = 1024). Right: truncated detector (m = 536).

The two-dimension (2D) spatial distribution of scattered x-ray photons of a complete detector and a truncated detector are shown in figure 10. The one-dimensional horizontal profile of the scatter and primary photon distributions were averaged over a rectangular region of interest (ROI) and over 30 projection views. The ROI starts from the 101st row and ends at the 200th row and is shown in figure 10.

**Figure 11.**
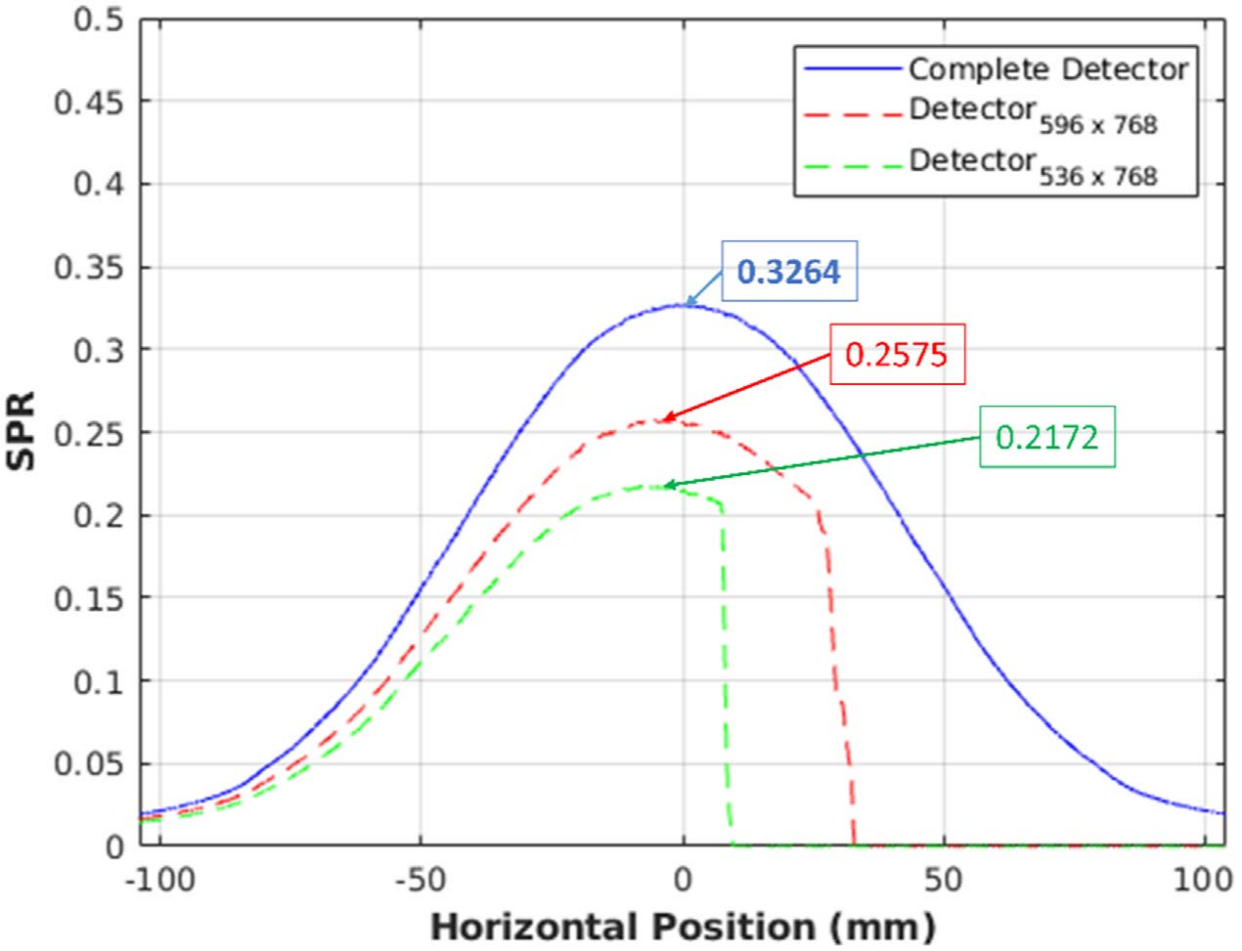
Horizontal SPR profile. Blue curve: The reference (1024 × 768 pixels complete detector). Red curve: 596 × 768 detector. Green curve: The detector of 536 × 768 pixels.

The maxima of the 1D horizontal SPR profile averaged over all projection views, ROI, and all thirty samples were 0.3264 ± $0.1245$, 0.2575 ± $0.1093$, and 0.2172 ± $0.083$ for the complete detector, 596- and 536-pixel detector, respectively (figure 11), resulting in 21.11% and 33.46% reduction in SPR. The MGD calculations were 8.3702 $\pm 1.9894$ mGy, 5.4604 ± $1.3686$, and 5.2022 ± $1.259$ mGy for the complete detector, 596- and 536-pixel detector, respectively, which indicates, 34.76% and 37.85% dose reduction.

## 4. Discussion

The traditional reconstruction algorithm, FDK with weighting schemes (FDK-W) in figure 4 and table 2, had degraded variance and SDNR when the number of pixels, *m*, of the detector along the fan-angle direction is decreased due to the less number of photons received by the trucated detector. The FDK-W method is a linear reconstruction algorithm that does not explicitly or implicitly account for image noise. In contrast, CS-based FRIST explicitly performs noise suppression through total variation-based regularization, and DL-based MS-RDN and AFN provide noise reduction through implicitly learning the distribution from the training data.

For the FDK-W reconstruction, artifacts can be observed when the number of pixels of the detector along the fan-angle direction, *m*, is less than 596. The CS-based algorithm, FRIST, and fully-trained deep-learning algorithm, MS-RDN, outperformed the FDK when *m* was larger or equal to 596. The AFN algorithm is a self-supervised DL algorithm and does not require training data. The incomplete sinograms were inpainted completely by the AFN algorithm followed by the FDK reconstruction and did not show artifacts when *m* was decreased to 536 and the image quality was maintained. All algorithms except MS-RDN had the ability to maintain the resolution of the calcification.

Both the SPR and radiation dose were reduced when the dimension of the detector along the fan-angle direction, *m*, decreased. When the *m* is larger than half of the original dimension (512 in this study), there is an extended SPR in the horizontal direction (right-hand side of the SPR profile in figure 11) which contributes to scatter effects on the other half breast. SPR and radiation dose can be reduced simultaneously using the truncated detector. The dose using the 596- and 536- detectors were 5.46 and 5.20 mGy, respectively, which are in the range of 3 to 6 mGy. Although the image quality of AFN with *m* = 536 and *m* = 596 are similar, the SPR decreases significantly when *m* decreases from 596 to 536.

One limitation of this study is that the truncated sinograms were retrospectively truncated from the clinical dataset acquired by the complete detector. Hence, the impact of x-ray scatter reduction could not be demonstrated in this study. Another limitation in this study was the approximation used in the number of iterations in the FRIST, where the optimal parameters were determined at a fixed *m* = 640 and applied to all truncations studied. Also, the MS-RDN algorithm was trained using a fixed *m* = 596. Identifying the optimal hyperparameters for FRIST at each truncation level and retraining MS-RDN for each truncation level may potentially improve performance.

## 5. Conclusions

We have demonstrated simultaneous reduction of SPR and radiation dose using nearly half-sized detectors. Among the advanced algorithms investigated here, our recently developed algorithm, AFN, can allow the most reduction of SPR and radiation dose using the narrowest detector without sacrificing the image quality. The AFN algorithm is self-supervised, hence extra training images are not required. The technology developed here can be extended to other cone-beam systems.

## Data Availability

No new data were generated in this study. The data that support the findings of this study are available upon reasonable request from the authors.

## References

[bpexadb8f1bib1] Bian J, Yang K, Boone J M, Han X, Sidky E Y, Pan X (2014). Investigation of iterative image reconstruction in low-dose breast CT. Phys. Med. Biol..

[bpexadb8f1bib2] Cho P S, Rudd A D, Johnson R H (1996). Cone-beam CT from width-truncated projections. Comput. Med. Imaging Graph.

[bpexadb8f1bib3] Cole E B, Campbell A S, Vedantham S, Pisano E D, Karellas A (2015). Clinical performance of dedicated breast computed tomography in comparison to diagnostic digital mammography (Abstract # SSA01-09). Annual Meeting & Scientific Assembly of Radiological Society of North America, RSNA 2015.

[bpexadb8f1bib4] FDA US (2002).

[bpexadb8f1bib5] Feldkamp L A, Davis L C, Kress J W (1984). Practical cone-beam algorithm. J. Opt. Soc. Am. A.

[bpexadb8f1bib6] Fu Z, Tseng H W, Vedantham S (2023). An Attenuation Field Network for Cone-Beam Breast CT with a Laterally-shifted Detector in Short Scan.

[bpexadb8f1bib7] Fu Z, Tseng H W, Vedantham S (2024). An attenuation field network for dedicated cone beam breast CT with short scan and offset detector geometry. Sci. Rep..

[bpexadb8f1bib8] Fu Z, Tseng H W, Vedantham S, Karellas A, Bilgin A (2020). A residual dense network assisted sparse view reconstruction for breast computed tomography. Sci. Rep..

[bpexadb8f1bib9] Ghazi P (2020). Reduction of scatter in breast CT yields improved microcalcification visibility. Phys. Med. Biol..

[bpexadb8f1bib10] Ghazi P, Fu G, Ghazi T, Kazanzides P, Iordachita I (2023). Narrow beam breast CT: proof-of-concept. Med. Phys..

[bpexadb8f1bib11] Hammerstein G R, Miller D W, White D R, Masterson M E, Woodard H Q, Laughlin J S (1979). Absorbed radiation dose in mammography. Radiology.

[bpexadb8f1bib12] Hendrick R E, Pisano E D, Averbukh A, Moran C, Berns E A, Yaffe M J, Herman B, Acharyya S, Gatsonis C (2010). Comparison of acquisition parameters and breast dose in digital mammography and screen-film mammography in the American college of radiology imaging network digital mammographic imaging screening trial. AJR Am J Roentgenol.

[bpexadb8f1bib13] Lindfors K K, Boone J M, Nelson T R, Yang K, Kwan A L C, Miller D F (2008). Dedicated breast CT: initial clinical experience. Radiology.

[bpexadb8f1bib14] Mettivier G, Russo P, Lanconelli N, Meo S L (2012). Cone-beam breast computed tomography with a displaced flat panel detector array. Med. Phys..

[bpexadb8f1bib15] O’Connell A, Conover D L, Zhang Y, Seifert P, Logan-Young W, Lin C-F L, Sahler L, Ning R (2010). Cone-beam CT for breast imaging: radiation dose, breast coverage, and image quality. AJR Am J. Roentgenol.

[bpexadb8f1bib16] O’Connell A M, Karellas A, Vedantham S (2014). The potential role of dedicated 3D breast CT as a diagnostic tool: review and early clinical examples. Breast J..

[bpexadb8f1bib17] Pautasso J J, Caballo M, Mikerov M, Boone J M, Michielsen K, Sechopoulos I (2023). Deep learning for x-ray scatter correction in dedicated breast CT. Med. Phys..

[bpexadb8f1bib18] Ramamurthy S, D’Orsi C J, Sechopoulos I (2016). X-ray scatter correction method for dedicated breast computed tomography: improvements and initial patient testing. Phys. Med. Biol..

[bpexadb8f1bib19] Rutt B, Fenster A (1980). Split-filter computed tomography: a simple technique for dual energy scanning. J. Comput. Assist. Tomogr..

[bpexadb8f1bib20] Schaefer D, Grass M, Haar P, van de (2011). FBP and BPF reconstruction methods for circular x-ray tomography with off-center detector. Med. Phys..

[bpexadb8f1bib21] Sechopoulos I (2012). X-ray scatter correction method for dedicated breast computed tomography. Med. Phys..

[bpexadb8f1bib22] Sechopoulos I, Bliznakova K, Qin X, Fei B, Feng S S J (2012). Characterization of the homogeneous tissue mixture approximation in breast imaging dosimetry. Med. Phys..

[bpexadb8f1bib23] Shi L, Vedantham S, Karellas A, O’Connell A M (2013). Technical note: skin thickness measurements using high-resolution flat-panel cone-beam dedicated breast CT. Med. Phys..

[bpexadb8f1bib24] Shi L, Vedantham S, Karellas A, Zhu L (2016). Library based x-ray scatter correction for dedicated cone beam breast CT. Med. Phys..

[bpexadb8f1bib25] Shi L, Vedantham S, Karellas A, Zhu L (2017). X-ray scatter correction for dedicated cone beam breast CT using a forward-projection model. Med. Phys..

[bpexadb8f1bib26] Sidky E Y, Pan X (2008). Image reconstruction in circular cone-beam computed tomography by constrained, total-variation minimization. Phys. Med. Biol..

[bpexadb8f1bib27] Sujlana P S, Mahesh M, Vedantham S, Harvey S C, Mullen L A, Woods R W (2019). Digital breast tomosynthesis: image acquisition principles and artifacts. Clin Imaging.

[bpexadb8f1bib28] Trinate R, Tseng H W, Larsen T, Fu Z, Vedantham S (2023). Objective Physics-Based Image Quality Characterization of a Low-Noise, High-Resolution, Dedicated Cone-Beam Breast CT Using an Offset Detector.

[bpexadb8f1bib29] Tseng H W, Fu Z, Vedantham S (2023a). Radiation dose Reduction in Cone-Beam Breast CT using Shorter x-ray Pulse-Width with a Self-Supervised Deep Learning Algorithm.

[bpexadb8f1bib30] Tseng H W, Karellas A, Vedantham S (2022). Cone-beam breast CT using an offset detector: effect of detector offset and image reconstruction algorithm. Phys. Med. Biol..

[bpexadb8f1bib31] Tseng H W, Karellas A, Vedantham S (2023b). Dedicated cone-beam breast CT: data acquisition strategies based on projection angle-dependent normalized glandular dose coefficients. Med. Phys..

[bpexadb8f1bib32] Tseng H W, Karellas A, Vedantham S (2021). Radiation dosimetry of a clinical prototype dedicated cone-beam breast CT system with offset detector. Med. Phys..

[bpexadb8f1bib33] Tseng H W, Karellas A, Vedantham S (2020a). Sparse-view, short-scan, dedicated cone-beam breast computed tomography: image quality assessment. Biomed. Phys. Eng. Express.

[bpexadb8f1bib34] Tseng H W, Vedantham S, Karellas A (2020b). Cone-beam breast computed tomography using ultra-fast image reconstruction with constrained, total-variation minimization for suppression of artifacts. Physica Med..

[bpexadb8f1bib35] Vedantham S, Karellas A (2018). Emerging breast imaging technologies on the horizon. Semin Ultrasound CT MR.

[bpexadb8f1bib36] Vedantham S, Shi L, Karellas A, Noo F (2012a). Dedicated breast CT: radiation dose for circle-plus-line trajectory. Med. Phys..

[bpexadb8f1bib37] Vedantham S, Shi L, Karellas A, O’Connell A M (2012b). Dedicated breast CT: fibroglandular volume measurements in a diagnostic population. Med. Phys..

[bpexadb8f1bib38] Vedantham S, Shi L, Karellas A, O’Connell A M, Conover D L (2013). Personalized estimates of radiation dose from dedicated breast CT in a diagnostic population and comparison with diagnostic mammography. Phys. Med. Biol..

[bpexadb8f1bib39] Vedantham S, Tseng H-W, Konate S, Shi L, Karellas A (2020). Dedicated cone-beam breast CT using laterally-shifted detector geometry: quantitative analysis of feasibility for clinical translation. J. X-Ray Sci. Technol..

[bpexadb8f1bib40] Wang G (2002). X-ray micro-CT with a displaced detector array. Med. Phys..

[bpexadb8f1bib41] Wienbeck S, Uhlig J, Luftner-Nagel S, Zapf A, Surov A, von Fintel E, Stahnke V, Lotz J, Fischer U (2017). The role of cone-beam breast-CT for breast cancer detection relative to breast density. Eur Radiol.

[bpexadb8f1bib42] Xie H, Shan H, Cong W, Liu C, Zhang X, Liu S, Ning R, Wang G (2020). Deep efficient end-to-end reconstruction (DEER) network for few-view breast CT image reconstruction. IEEE Access.

